# Data on the critical condition of silica and ice particles removal from surface

**DOI:** 10.1016/j.dib.2020.105363

**Published:** 2020-02-29

**Authors:** Zheyuan Liu, Qingping Li, Jin Fu, Mingjun Yang, Jiafei Zhao

**Affiliations:** a Key Laboratory of Ocean Energy Utilization and Energy Conservation of Ministry of Education, Dalian University of Technology, Dalian, 116024, China; b CNOOC Research Institute Co. Ltd., China

**Keywords:** Particle removal, Surface, Liquid bridge, Adhesion

## Abstract

Data on particle removal from surfaces is yet to be presented properly. This data is explored and the mathematical models are presented in the previous paper “New model for particle removal from surface in presence of deformed liquid bridge” [1], which predict the fluid velocity required to initiate the motion of a particle. However, the models still need to be verified by the experiment. The experimental data in this paper measured the critical fluid flow velocity when the particles were about to removal from the surface. The particle removal including the process without the effect of liquid bridge and the process with the existence of liquid bridge. Different diameter of the silica particles were used to measured the critical fluid flow velocity without the liquid bridge. In addition, with the existing of the liquid bridge, the same diameter of the silica particles and the ice particles were used to researched the critical state. The data has implications in furthering the understanding of the underlying mechanisms during the removal of particles from surfaces exposed to fluid flow.

**Table:** Specifications Table

Subject	Physics and Astronomy; Surfaces and Interfaces
Specific subject area	Particle removal and adhesion
Type of data	Table Figure
How data were acquired	The experiment data were acquired by the particle removal parameters measurement device. The gas flow velocity values were measured by the gas flow meter (Sierra, USA, measurement range: 0–700 m³/h, measurement accuracy: ±0.1%). The contact angles were measured by the CCD camera.
Data format	Raw Analyzed
Parameters for data collection	For collection data: Critical fluid bulk velocity, Particle radius, Liquid bridge length, Embracing angle, Contact angle, Front angle, Rear angle and Liquid bridge volume.
Description of data collection	For the experiments in presence of liquid bridge, a small amount volume of liquid was dropped on the surface. And the particle was placed embraced by the liquid droplet. The critical flow velocity was measured when the particle was about to removal from surface with the fluid flow velocity increasing gradually. The critical state of the particle was photographed by the CCD camera.
Data source location	Institution: Key Laboratory of Ocean Energy Utilization and Energy Conservation of Ministry of Education in Dalian University of Technology, No.2 Linggong Road, Ganjingzi District. City: Dalian Country: China
Data accessibility	With the article.
Related research article	Zheyuan Liu, Jin Fu, Mingjun Yang, Jiafei Zhao and Yongchen Song. “New model for particle removal from surface in presence of deformed liquid bridge.” *Journal of Colloid and Interface Science*, 2020 (562):268–272. https://doi.org/10.1016/j.jcis.2019.11.117

**Table:** 

**Value of the Data** • The data could verify the model for particles removal from the surface, even the particles in presence of the deformed liquid bridge. • The data could help explaining the different forms of the detachment between particles, liquid bridge and surface. It would further improve the mathematical model. • The data may be useful for other groups working or studying on the particle removal process. And making the researched further used in microelectronics, pneumatic conveying, aviation and flow assurance fields.

## 1 Data description

This section performs the experiment data in particle removal process, including the particle removal without liquid bridge and the particle removal in presence of deformed liquid bridge. The model for predicting the particle removal process has been studied in the earlier paper [1]. In this paper, the parameters which are associated with the data should be listed and some basic parameter (including fluid density, particle density, interfacial tension, fluid dynamic viscosity, fluid kinematic viscosity, liquid bridge shape parameter and friction coefficient) values need to be ensured in the experiments. Table 1 shows the nomenclatures with units and the exact values in the experiments.

**Table 1:** The nomenclatures and the values in the experiments.

Nomenclature		Unit	Values in the experiment
*r*_*p*_	Particle radius	m	/
*L*	Liquid bridge length	m	/
*α*	Embracing angle	°	/
*θ*_*p*_	Contact angle	°	/
*θ*_*front*_	Front angle	°	/
*θ*_*rear*_	Rear angle	°	/
*ρ*_*f*_	Fluid density	kg/m^3^	N_2_:1.23(0 °C); 1.15(20 °C)
*ρ*_*p*_	Particle density	kg/m^3^	Silica:2650; Ice:900
*U*	Fluid bulk velocity	m/s	/
*γ*_*l*_	Interfacial tension	N/m	Water:0.0755(0 °C); 0.0727(20 °C)
*μ*	Fluid dynamic viscosity	N·s/m^2^	N_2_: 1.66 × 10^−5^(0 °C); 1.76 × 10^−5^(20 °C)
*ν*	Fluid kinematic viscosity	m^2^/s	N_2_: 1.35 × 10^−5^(0 °C); 1.53 × 10^−5^(20 °C)
*Re*	Reynolds number	/	Silica:5155∼9860; Ice:8291∼13004
*Ca*	Capillary number	/	Silica:8.28 × 10^−4^∼1.22 × 10^−3^; Ice:8.20 × 10^−4^∼1.26 × 10^−3^
*k*	Liquid bridge shape parameter	/	Range:1/2∼π/2; *k* = 1(in the experiment)
*f*_*f*_	Friction coefficient	/	*f*_*f*_ = 0.2

**Fig. 1:**
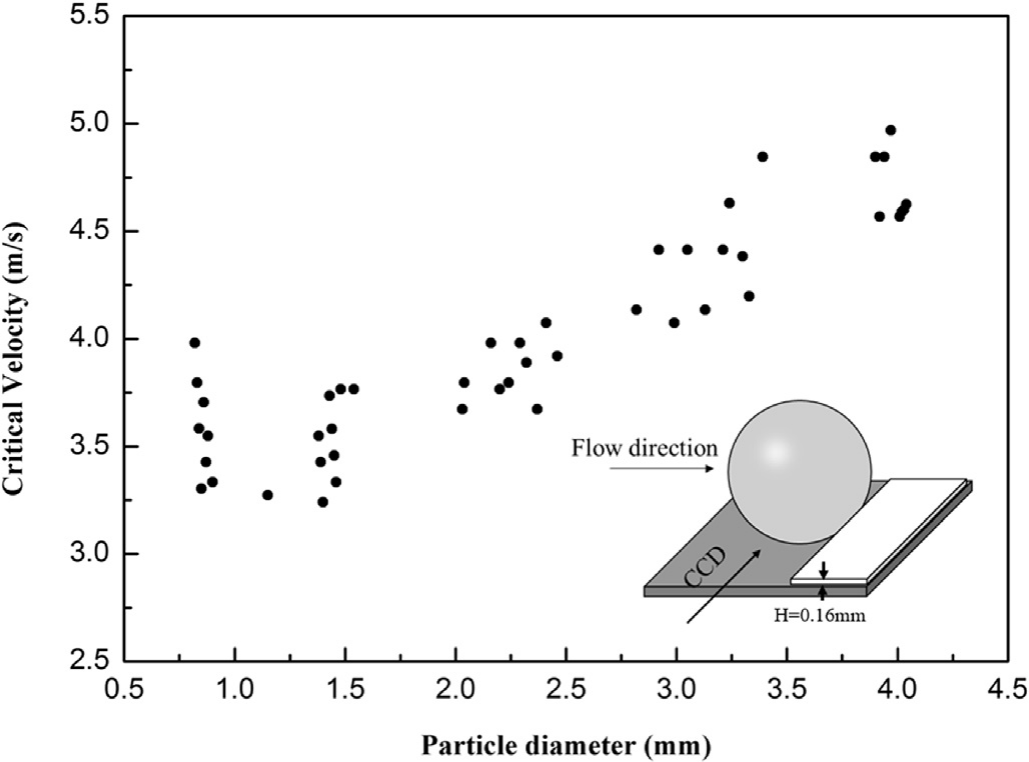
The changing of critical velocity with different particle diameter.

The experiment data in Fig. 1 shows the different critical flow velocity with different silica particle diameters without the liquid bridge [[2], [3], [4]]. In the experiment, the data of critical velocity were measured by the gas flow meter when the different diameters of particles (range: 0.82mm–4.04mm) were about to blow away from the surface. The experiment added a slice in front of the particle and the height of the slice was 0.16mm. This operation provided a specific resisting moment. The slice is equivalent to a resistance moment [3].

Table 2 and Table 3 show the experiment data about the silica particles and ice particles removal from the surface in presence of deformed liquid bridge at the critical condition [5,6]. The silica particle experiments measured the critical fluid bulk velocity, particle radii, the embracing angles and contact angles of the liquid bridge. The ice particle experiments measured the critical fluid bulk velocity, particle radii, liquid bridge length, the front angle and rear angle of the deformed liquid bridge [7,8].

**Table 2:** The silica particle contact angles and embracing angles at critical condition.

Liquid bridge volume (μL)	*r*_*p*_ (mm)	*θ*_*p*_	*α*	*U*(m/s)
0.5	2	54°	15°	3.456
1.0	2	45°	17°	3.426
1.5	2	42°	23°	3.642
2.0	2	33°	30°	3.766
2.5	2	23°	28°	3.827
3.0	2	22°	36°	3.858
3.5	2	27°	30°	4.506
4.0	2	41°	33°	4.383
4.5	2	42°	38°	4.136
5.0	2	25°	36°	4.445
5.5	2	16°	37°	4.138
6.0	2	26°	35°	4.198
6.5	2	20°	37°	4.506
7.0	2	23°	39°	4.383
7.5	2	28°	39°	4.846
8.0	2	16°	38°	4.753
8.5	2	34°	35°	4.756
9.0	2	21°	42°	4.815
9.5	2	20°	39°	4.661
10.0	2	17°	41°	5.031

**Table 3:** The ice particle front angles, rear angles and the liquid bridge length along the surface at critical condition.

Liquid bridge volume (μL)	*r*_*p*_ (mm)	*θ*_*front*_	*θ*_*rear*_	*L* (mm)	*U*(m/s)
0.5	2.177	116°	60°	3.323	3.735
1.0	2.311	110°	61°	3.204	3.881
1.5	2.451	108°	58°	3.545	4.599
2.0	2.393	116°	61°	3.544	4.661
2.5	2.451	116°	58°	4.246	4.691
3.0	2.089	113°	57°	4.186	4.877
3.5	2.225	117°	53°	4.346	5.031
4.0	1.961	120°	57°	4.345	5.185
4.5	2.155	121°	59°	4.321	5.371
5.0	2.215	126°	55°	4.597	5.432
5.5	2.223	127°	56°	4.466	5.253
6.0	2.253	128°	57°	4.349	5.185
6.5	2.175	128°	57°	4.446	5.188
7.0	2.069	139°	45°	4.575	5.463
7.5	2.074	133°	47°	4.852	5.371
8.0	1.853	128°	48°	4.958	5.709
8.5	2.343	128°	51°	5.304	5.494
9.0	2.377	115°	60°	5.617	5.525
9.5	2.301	124°	55°	5.518	5.339
10.0	2.232	118°	51°	5.838	5.667

## 2 Experimental design, materials, and methods

**Fig. 2:**
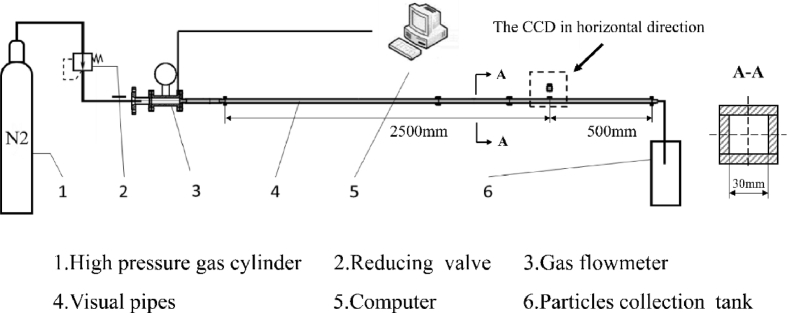
The experiment device for the fluid bulk velocity measurement in the particle removal process.

This section introduces the experimental design and methods in the earlier paper [1]. The model for particle removal was presented and the model also need to be verified by the experiment. The experiment device is shown in Fig. 2. The nitrogen gas cylinder is connected with a reducing valve. The gas flow meter (Sierra, USA, measurement range: 0–700 m³/h, measurement accuracy: ±0.1%) is installed after the valve. A visual tube, which is made of acrylic material, is connected after the gas flow meter. The length of the tube is 3 m and the diameter of the rectangular tube is *d* = 30mm. The distance between the particles placed position and the inlet is *l* = 2.5 m which keeps the fluid in this parts stable (*l/d* > 50∼60). The experiment measured the fluid bulk velocity in the visual square tube by gas flowmeter and collected the pictures by the CCD cameras. The experiment device is settled in a thermostatic chamber which can control the environment temperature.

The gas fluid in the experiment was nitrogen with the purity of 99.999%. The particles in the experiment were silica particles and ice particles. The silica particles (AS-ONE company in Japan) were standard spherical. The experiment used four diameter range of the silica particles including 0.82∼1.54mm, 2.03∼2.46mm, 2.82∼3.39mm and 3.90∼4.04mm. The ice particles were made by the liquid droplets and the liquid nitrogen. The liquid droplets were dropped into the liquid nitrogen with a injector. The liquid droplets would form ice particles rapidly due to the low temperature. Then, the ice particles should be taken out of the liquid nitrogen as soon as possible to avoid the split. The radii of the ice particles were listed in Table 3.

For measuring the critical fluid bulk velocity in particle removal process, the particles (silica or ice) should be placed on the observation position of the surface. For the experiment with the liquid bridge, different volume of the liquid should be dropped on the surface by the injector before placing the particles. Then, adjusted the reducing valve to make the fluid flow velocity increased gradually. At the same time, the state of the particles could be observed by the CCD camera. When the particles removal from the surface, the critical flow velocity were recorded. The pictures of the particles in critical state were analyzed to obtain the contact angles.
